# Was there a weekend effect on mortality rates for hospitalized patients with COVID-19 and acute myocardial infarction? Insights from the National Inpatient Sample, 2020

**DOI:** 10.25122/jml-2023-0341

**Published:** 2024-03

**Authors:** Sai Priyanka Mellacheruvu, Sai Prasanna Lekkala, Puneeteshwar Singh Khela, Gurjot Singh, Karanvir Singh Gill, Utsav Premjibhai Vaghani, Sahas Reddy Jitta, Mohmed Junaid Hingora, Manali Patel, Sriharsha Dadana, Rupak Desai

**Affiliations:** 1 Department of Public Health, University of Massachusetts, Lowell, USA; 2 Department of Internal Medicine, Mamata Medical College, Khammam, India; 3 Department of Internal Medicine, Sri Guru Ram Das Institute of Medical Sciences and Research, Amritsar, India; 4 Department of Internal Medicine, Smt. N.H.L. Municipal Medical College, Ahmedabad, India; 5 Department of Internal Medicine, Mercy Hospital, St Louis, Missouri, USA; 6 Department of Internal Medicine, Pandit Deendayal Upadhyay Medical College, Rajkot, India; 7 Department of Internal Medicine, Gastroenterology and Internal Medicine Specialists, Lake Barrington, Illinois; 8 Department of Hospital Medicine, Cheyenne Regional Medical Center, Cheyenne, USA; 9 Independent Researcher, Atlanta, GA, USA

**Keywords:** COVID-19/SARS-CoV-2, coronavirus, acute myocardial infarction, weekend effect, mortality, AMI, Acute Myocardial Infarction, NIS, National Inpatient Sample, OR, Odds Ratio, HCUP, Healthcare Cost And Utilization Project, CAD, Coronary Artery Disease, ARDS, Acute Respiratory Distress Syndrome

## Abstract

Our study aimed to assess the effect of weekend versus weekday hospital admissions on all-cause mortality in patients with acute myocardial infarction (AMI) and COVID-19 during the COVID-19 pandemic. We analyzed data from the National Inpatient Sample (NIS) 2020, identifying patients with co-existing AMI and COVID-19 admitted on weekdays and weekends. Baseline demographics, comorbidities, and outcomes were assessed. A multivariable regression analysis was conducted, adjusting for confounders to determine the odds of all-cause mortality. Among 74,820 patients, 55,145 (73.7%) were admitted on weekdays, while 19,675 (26.3%) were admitted on weekends. Weekend admissions showed slightly higher proportions of men (61.3% vs. 60%) and whites (56.3% vs. 54.9%) with a median age of 73 years (range: 62-82). The overall all-cause mortality had an odds ratio (OR) of 1.00 (95% CI, 0.92–1.09; *P* = 0.934). After adjusting for covariates, there was no significant associations between mortality and hospital type (rural: OR = 1.04; 95% CI, 0.78–1.39; *P* = 0.789; urban teaching: OR = 1.04; 95% CI, 0.94–1.14; *P* = 0.450) or geographic region (Northeast: OR = 1.16; 95% CI, 0.96–1.39; *P* = 0.12; Midwest: OR = 0.99; 95% CI, 0.83–1.17; *P* = 0.871; South: OR = 0.97; 95% CI, 0.85–1.12; *P* = 0.697; West: OR = 0.94; 95% CI, 0.77–1.15; *P* = 0.554). There was no significant difference in the rate of all-cause mortality among patients admitted for AMI and COVID-19 between weekdays and weekends.

## INTRODUCTION

The 'weekend effect' refers to the higher mortality rates observed in patients hospitalized on weekends compared to weekdays. In a decade-long study, Handel *et al*. [1] showed increased mortality on weekends for many diagnoses, including acute myocardial infarction (AMI). Similarly, another study showed increased mortality among patients presenting with AMI on weekends [2]. There could be several possible explanations for this weekend effect, both intrinsic and extrinsic, to the patient [3]. Extrinsic factors potentially contributing to higher mortality rates include reduced weekend staffing and delays in obtaining necessary procedures. Intrinsic factors related to the patient may include the severity of their illness and the burden of comorbidities [3]. It is well-reported in the literature that patients with COVID-19 have an increased risk of AMI [4]. However, no research has examined the weekend effect on mortality among COVID-19 patients with AMI. Understanding potential variations in outcomes and healthcare delivery based on admission day is crucial. Therefore, this study aimed to compare mortality rates among COVID-19 patients with AMI admitted on weekends versus weekdays.

## MATERIAL AND METHODS

The National Inpatient Sample (NIS), 2020, a publicly available database from the Healthcare Cost and Utilization Project (HCUP) sponsored by the Agency for Healthcare Research and Quality, contains data from over 35 million hospitalizations per year from non-federal acute care hospitals in 45 states [5]. For this study, we included data from January 2020 to December 2020. We identified patients who were diagnosed with COVID-19 and AMI at the time of admission using ICD-10 codes U0711 for COVID-19 and code 122 for AMI. These patients were categorized into weekend (Saturday and Sunday) and weekday (Monday to Friday) admissions. Additionally, we assessed baseline demographics, hospital-level characteristics, and associated comorbidities. The primary objective was to assess the impact of weekend versus weekday hospital admissions on all-cause mortality among patients diagnosed with AMI and COVID-19 during the COVID-19 pandemic 2020. We also assessed baseline demographic factors, and the prevalence and associated mortality of underlying critical illnesses. The study analyzed the mortality rate in weekend vs. weekday admissions using a propensity score-matched analysis. The analysis included a multivariate logistic regression model adjusted for age, sex, race, and comorbidities. All statistical analyses were performed using SPSS Statistics software, with P ≤0.05 considered significant (Table 1).

**Table 1:** Baseline comorbidities and outcomes in weekday vs. weekend AMI admissions with COVID-19, 2020

VARIABLES		Weekday vs. Weekend		
		Weekday	Sat/Sun	*P* value
		Column %		
Age in years at admission	Median [IQR]	72 [62-83]	73 [63-82]	0.001
Sex	Male	61.3%	60.0%	0.001
	Female	38.7%	40.0%	
Race	White	56.3%	54.9%	0.003
	Black or African American	19.6%	19.7%	
	Hispanic	19.4%	20.2%	
	Asian/Pacific Islander	3.7%	4.1%	
	Native American	1.1%	1.1%	
Median household income national quartile for patient ZIP Code	1	35.7%	36.2%	<0.001
	2	28.4%	28.8%	
	3	21.5%	19.5%	
	4	14.5%	15.5%	
Payor type	Medicare	70.1%	69.8%	0.175
	Medicaid	10.1%	10.6%	
	Private	16.7%	16.7%	
	Self-pay	2.9%	2.7%	0.175
	No Change	0.2%	0.2%	0.175
Elective	Non-elective	97.1%	97.8%	<0.001
	Elective	2.9%	2.2%	<0.001
Hospital location & teaching status	Rural	8.4%	8.0%	0.025
	Urban Nonteaching	17.4%	18.1%	
	Urban Teaching	74.2%	73.9%	
Hospital region	Northeast	20.2%	19.5%	<0.001
	Midwest	21.1%	19.2%	
	South	24.1%	22.9%	
	West	37.2%	38.9%	
**COMORBIDITIES:**				
Hypertension		78.9%	79.7%	0.028
Diabetes		48.5%	49.3%	0.036
Hyperlipidemia		49.3%	49.6%	0.495
Obesity		21.3%	22.0%	0.054
Peripheral vascular disease		6.6%	7.7%	<0.001
Tobacco use disorder		21.3%	21.4%	0.694
Prior MI		8.9%	8.9%	0.795
Prior VTE		4.0%	3.2%	<0.001
Cancer		4.5%	4.4%	0.861
Chronic kidney disease		38.0%	37.6%	0.394
Acquired immune deficiency syndrome		0.6%	0.6%	0.371
Alcohol abuse		2.7%	1.7%	<0.001
Drug abuse		2.0%	2.3%	0.008
Cannabis use disorder		0.6%	0.5%	0.246
Depression		9.2%	9.8%	0.015
Chronic pulmonary disease		24.0%	22.4%	<0.001
Hypothyroidism		13.3%	14.1%	0.005
Other thyroid disorders		1.2%	0.8%	<0.001
Valvular disease		1.7%	1.8%	0.286
OSA		7.5%	7.3%	0.283
Prior PCI		0.8%	0.9%	0.291
Prior TIA stroke		7.7%	8.0%	0.146
Prior chemo		0.7%	0.6%	0.11
Prior radiation		0.8%	1.0%	0.009
**OUTCOMES:**				
Death during hospitalization		35.5%	36.0%	0.228
Disposition of patient (uniform)	Routine	24.4%	25.2%	<0.001
	Transfer to Short-Term Hospital	3.3%	3.0%	
	Transfer Other: SNF, ICF, etc.	24.4%	23.1%	
	Home Health Care	11.4%	11.6%	
Cardiogenic shock		4.7%	4.8%	0.424
Health care charges (Cost)		$168736.2	$166709.1	0.694
Length of stay (days)		10.752467	10.656414	0.021

MI, Myocardial infarction; VTE, Venous Thromboembolism; SNF, Skilled Nursing Facility; ICF, Intermediate Care Facility; IQR-Inter-quartile range; OSA, Obstructive Sleep Apnea; PCI, Percutaneous Coronary Intervention; TIA, Transient Ischemic Attack.

*P* <0.05 was considered statistically significant.

## RESULTS

A total of 74,820 patients with AMI and COVID-19 were included in the study. Of these, 55,145 (73.7%) were admitted on weekdays and 19,675 (26.3%) on weekends. Weekend admissions had a higher percentage of male participants (61.3% vs. 60%) and white individuals (56.3% vs. 54.9%), with a median age of 73 years in both cohorts. In addition, the weekend cohort also had a higher prevalence of diabetes (49.3% vs. 48.5%), hypertension (49.3% vs. 48.5%), peripheral vascular disease (7.7% vs. 6.6%), and hypothyroidism (14.1% vs. 13.3%) (all *P* < 0.05). The overall all-cause mortality was 1.00 (95% CI, 0.92–1.09; *P* = 0.934). After adjusting for covariates, there was no significant association with all-cause mortality in rural (OR = 1.04; 95% CI, 0.78–1.39; *P* = 0.789) and urban teaching hospitals (OR = 1.04; 95% CI, 0.94–1.14; *P* = 0.450) compared to urban non-teaching hospitals (OR = 0.86; 95% CI, 0.70–1.05; *P* = 0.141). Regional analysis showed that the Northeast (OR = 1.16; 95% CI, 0.96–1.39; *P* = 0.12) also had no significant association with all-cause mortality compared to the Midwest (OR =0.99; 95% CI, 0.83–1.17; *P* = 0.871), South (OR = 0.97; 95% CI, 0.85–1.12; *P* = 0.697), and West (OR = 0.94; 95% CI, 0.77–1.15; *P* = 0.554) (Table 2). No statistically significant difference was observed between healthcare costs among weekend vs. weekday admissions ($168736.2 vs. 166709.1; *P* = 0.694). Furthermore, the length of stay was similar in both cohorts (10.7 vs. 10.6, *P* = 0.021).

**Table 2:** Adjusted multivariable odds of all-cause mortality in weekend vs. weekday AMI admissions with COVID-19

All-cause mortality	Odds Ratio	95% CI		*P* value
		Lower	Upper	
Dependent all-cause mortality	1.00	0.92	1.09	0.934
**Sub-population: hospital location & teaching status**				
Rural	1.04	0.78	1.39	0.789
Urban Nonteaching	0.86	0.70	1.05	0.141
Urban Teaching	1.04	0.94	1.14	0.450
**Sub-population: hospital region**				
Northeast	1.16	0.96	1.39	0.122
Midwest	0.99	0.83	1.17	0.871
South	0.97	0.85	1.12	0.697
West	0.94	0.77	1.15	0.554

Multivariable logistic regression was adjusted for sex, age, race, median household income, hospital location, payer type, and admission type (weekend or weekday), and comorbidities like diabetes, hyperlipidemia, obesity, tobacco use disorder, prior myocardial infarction, prior stroke, prior venous thromboembolism, prior coronary artery bypass grafting, cancer, chronic kidney disease, alcohol use, drug abuse, chronic lung diseases, valvular disorders, and cannabis use disorder. C-statistics > 0.07. A two-tailed P <0.05 was considered significant.

## DISCUSSION

Coronary artery disease (CAD) is among the leading causes of mortality, causing close to 9 million deaths annually [6]. According to a 2016 American Heart Association update, CAD causes over 500,000 deaths annually in the United States alone [7]. The effectiveness of treatment greatly depends on timely intervention, with particular emphasis placed on reducing the 'door to balloon' time [7,8]. The mortality rate for AMI increased significantly from 5.2% pre-COVID-19 to 17.7% during the COVID-19 pandemic [9]. COVID-19 is an independent risk factor for in-hospital mortality in AMI patients, with some studies showing that this risk is higher in patients with pre-existing cardiovascular diseases, who are more susceptible to myocardial injury [10-12]. The severity of presentation was more pronounced during the pandemic, with increased levels of cardiac enzymes, reduced left ventricular ejection fraction, and a 25% increase in the need for inotropic support due to critically ill cardiac patients [9]. The time from symptom onset to first medical contact was prolonged in all AMIs during the pandemic, with studies showing that the time from symptom onset to coronary angiography increased by 39.2% due to disruptions in workflow [9,13]. Additionally, patients with COVID-19 and AMI had other complications related to COVID-19, such as bilateral pneumonia, the need for mechanical ventilation because of acute respiratory distress syndrome (ARDS), and higher mortality than patients without ARDS [10].

A study by Kostis *et al*. [14] showed that weekend admissions for myocardial infarction had higher mortality rates than weekdays because of decreased access to invasive cardiac procedures. Over the past 15 years, there has been increasing attention regarding the risks associated with weekend mortality among in-hospital admissions. A study by Munshi *et al*. [15] with a sample size of 2,206,289 myocardial infarction admissions found a higher mortality rate on weekends compared to weekdays, with adjusted odds ratios (aOR) of 1.15 (*P* <0.01). Supporting these findings, a study by Khoshchehreh *et al*. [16] reported that weekend AMI admissions were associated with poorer revascularization rates and longer intervention times than weekday admissions [16]. However, a recent national analysis covering the period from 2000-2008 revealed that implementing standardized protocols and more widespread use of cardiac catheterization significantly reduced in-hospital AMI mortality rates from 9.4% in 2000 to 7.1% in 2008. This analysis also showed that the weekend mortality effect previously observed had largely disappeared by 2006 [17].

Our study also demonstrated no significant differences in mortality rates among patients with AMI and COVID-19, irrespective of time of admission. Our findings agree with recent studies by Noad *et al*. and Vallabhajosyula *et al*., which showed no significant association between weekend effects and in-hospital mortality [18,19]. Another large-scale study by Rattka *et al*. [20] in Germany among patients with AMI and COVID-19 also reported no significant increase in in-hospital mortality rates during the pandemic. This consistency is likely due to the standardized treatment protocols for AMI, which maintain high-quality care regardless of when symptoms occur, and these protocols were effectively implemented throughout the pandemic.

In contrast, a meta-analysis by Yu *et al*. [21] noted that out-of-hour admissions (weekends and holidays) may be associated with an increased risk of both short- and long-term mortality in patients with AMI [21]. While contradictory findings are reported in this study, the authors elaborate that this meta-analysis included studies from both developing and developed countries, which may not apply to all included countries due to varied treatment protocols.

Furthermore, our study found no significant difference in mortality rates between weekend and weekday admissions for patients with AMI and COVID-19. This finding was consistent across rural and urban teaching hospitals, geographic regions (Northeast US), and rural and urban non-teaching hospitals. In contrast, a study by Loccoh *et al*. showed increased AMI mortality in rural hospitals compared to urban hospitals [22]. Similarly, Mekonnen *et al*. [23] found a higher mortality rate for patients hospitalized on weekends (8.5% vs. 7.4%) and a more substantial weekend impact in rural hospitals than in urban hospitals. The findings indicate a deficiency in life-saving procedures and reduced care accessibility for those receiving them on weekends, resulting in less intensive medical care. Other possible explanations include decreased staffing and increased physician responsibility in rural settings [17,18].

Along with extrinsic factors related to staffing, another plausible explanation for the increase in weekend hospital mortality could be the result of a skewed population of severely ill patients admitted on weekends. According to our research, weekend admissions had a higher prevalence of baseline comorbidities than weekday admissions, including hypertension, diabetes, peripheral vascular disease, depression, drug misuse, and thyroid problems. Noad *et al*. [19] reported similar findings. Patients with advanced or symptomatic conditions, who might have delayed seeking medical care to avoid missing work, may present on the weekends. In contrast, those with less severe conditions may delay admission [16]. In addition to chronic comorbidities management, access to medical care was also impacted during COVID-19, with approximately 41% of US adults delaying or avoiding medical care, including urgent or emergent medical care during the pandemic. There has also been a significant reduction in elective cardiac procedures such as angiographies and valve replacements, thus impacting the overall cardiovascular health of the population [24-26]. These findings highlight an essential aspect regarding healthcare delivery and patient outcomes related to COVID-19 and AMI within hospital settings. The decrease in overall AMI-related mortalities is undoubtedly commendable; however, vigilance must be maintained when evaluating disparities between different periods and days of the week. Understanding variations in healthcare delivery, including disparities across weekdays and weekends, can guide interventions to improve equitable access and consistent quality care for patients with AMI and similar conditions requiring immediate medical attention.

Our study has several limitations. This retrospective cohort study used ICD-10 codes for AMI, COVID-19, and baseline characteristics. However, considering the nature of the study, a prospective study is not feasible. We cannot randomize patients into weekend vs. weekday admissions. Coding practices may vary across various hospitals. The NIS tracks hospital discharges rather than individual patients. This limitation prevented us from identifying patients readmitted during the study period. The NIS data did not distinguish between weekdays and national holidays in its data marking. Federal holidays falling on weekdays were treated as regular weekdays. This study examined deaths that occurred during the hospitalization period.

## CONCLUSION

In conclusion, our study found no significant difference in overall mortality for patients with AMI and COVID-19 admitted on weekends compared to weekdays. However, complications associated with COVID-19, limited resources, and access to revascularization procedures can cause increased weekend mortality. Considering this, healthcare policies, organizations, and clinicians should carefully allocate their resources over the weekend to decrease mortality rates associated with COVID-19 and AMI admissions. The discrepancy in care delivery among rural hospitals increased, especially during the COVID-19 pandemic. Limited weekend staffing, resources, and physician workload contribute to this disparity. Developing effective interventions and policies to ensure consistent, high-quality care for patients with AMI and COVID-19 in rural and urban settings requires a comprehensive understanding of resource allocation and patient-related factors. This knowledge is essential for tailoring healthcare services to meet the specific needs of these individuals throughout the week.

## Data Availability

All data generated or analyzed during this study are included in this published article. Further data is available from the corresponding author upon reasonable request.
